# Evaluating the sustainability of lightweight drones for delivery: towards a suitable methodology for assessment

**DOI:** 10.1186/s42252-023-00040-4

**Published:** 2023-04-20

**Authors:** Sinéad Mitchell, Juliana Steinbach, Tomás Flanagan, Pouyan Ghabezi, Noel Harrison, Simon O’Reilly, Stephen Killian, William Finnegan

**Affiliations:** 1 University of Galway; J.E. Cairnes School of Business and Economics; School of Engineering, Ryan Institute, University Road, Galway, Ireland; 2 I-Form, the SFI Research Centre for Advanced Manufacturing, UCD, Dublin, Ireland; 3 Engineering Department ÉireComposites Teo, Inverin, Co Galway Ireland; 4 Manna Drone Delivery Ltd, NovaUCD, UCD, Belfield Innovation Park, Dublin, Ireland

**Keywords:** Drone delivery, LCA, Sustainability, Composite, Manufacturing

## Abstract

Drone technology is widely available and is rapidly becoming a crucial instrument in the functions of businesses and government agencies worldwide. The demand for delivery services is accelerating particularly since the Covid-19 pandemic. Both companies and customers want these services to be efficient, timely, safe, and sustainable, but these are major challenges. Last-mile delivery by lightweight short-range drones has the potential to address these challenges. However, there is a lack of consistency and transparency in assessing and reporting the sustainability of last-mile delivery services and drones. This paper critically reviews published papers on Life Cycle Assessments of drones to date. The study reveals a lack of comprehensive studies, and a need to examine composite and battery manufacturing developments and provides key considerations for future study development.

**Graphical Abstract:**

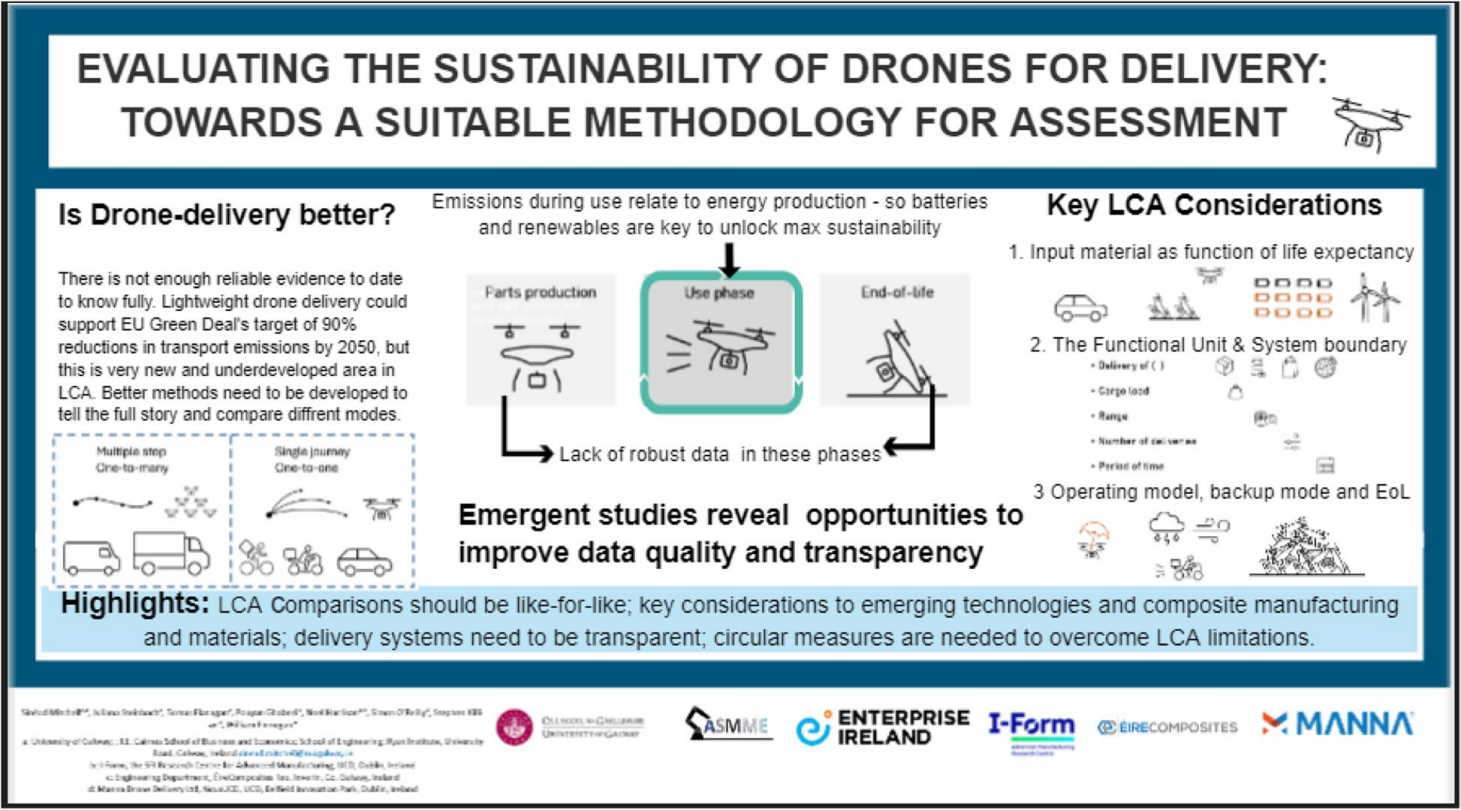

## Introduction

The climate crisis is gathering increasing attention with persistent reports of devastating events such as wildfires, flooding, drought, and storms. A stark warning from the Intergovernmental Panel on Climate Change (IPCC) is calling for urgent actions in all sectors, putting into doubt the target of limiting global warming below 1.5 °C [1]. The transport sector is responsible for 25% of net anthropogenic GHG emissions in the EU [2] and 15% globally [1]. The EU Green Deal aims for a 90% reduction in emissions from transport by 2050 [3]. Mitigation actions in the transport sector come with co-benefits of cleaner air, reduced traffic congestion, and reduced material demand [1] as well as a reduced carbon tax on fossil fuel free transport options. In Ireland the transport sector is the fastest-growing source of emissions, increasing by 100% since 1990, representing 18% of GHG emissions and 90% attributed to road transport [4]. Ireland’s climate action plan aims to reduce emissions by 42–50% to transport modes with lower energy consumption [5].

This study is focused on one specific aspect of transport: Unmanned Aerial Vehicles (UAVs) otherwise known as drones. Drones are a relatively new transport technology, and a rapid increase in delivery services is expected [6]. Drones for delivery could displace some road-dominated delivery modes (mainly cars and vans) and contribute to emission reduction targets. However, the technology is in its infancy and there is a dearth of knowledge on environmental impacts. New disruptive technologies should consider sustainability aspects for future scenarios as companies plan to scale the production and use of drones [7]. Future designs should align with the European Green Deal for industry [8], the EU Circular Economy Action Plan (CEAP) [9], and the Sustainable Development Goals (SDGs). Composite materials play a key part in reducing the weight of aerospace materials, which in turn helps to reduce fuel, energy, and emissions. However, most composites are not widely recycled or designed with sustainability considerations.

There is currently no agreed best practice methodology among the scientific community to conduct sustainability assessments of drones or drone delivery systems, particularly parts production of drones or drone delivery services. There has been little scrutiny of the composite material contribution to date, but there are many benefits to replacing traditional materials with composites to make lighter more efficient airframes. EU and country action plans are resulting in a shift towards more sustainable materials. There are many issues to consider related to composites such as the material inputs, the manufacturing processes, and the difficulty with recycling composites as most airframes of drones are not currently designed with disassembly, recovery, or reuse of the materials in mind.

This paper explores the sparse but growing body of research in understanding the sustainability of lightweight drones and drone delivery services. There is currently no agreed best practice methodology and there has been little scrutiny of the parts production and composite material contribution to date. A clear and robust methodology to develop impact models is crucial in understanding the complexity of a comparative assessment. A critical analysis of current studies to assess the sustainability of drones and drone services is examined and appropriate methodology considerations to assess lightweight drones and drone delivery systems are made.

This work is part of the Mi-Drone project funded by Enterprise Ireland Disruptive Technology Innovation Fund (DTIF) Grant no. DT 2020 0221. The project is developing a new lightweight drone through a joint industry and academic research collaboration. The Mi-Drone project aspires to develop the technology to scale up manufacturing to align with more sustainable drone services to be operationalised in the fast-food, pharmacy, and the grocery delivery.

## Methodology

### Aim and research questions

This paper seeks to synthesise emerging studies on the sustainability assessments of drone technologies, including the drone itself (as a product) and the delivery service a drone provides (as a product system). The study involved a review of the select body of literature, as a full systematic review was deemed unfitting because there are very few targeted studies. A critical analysis of the literature will describe the main findings with the main focus on methodological approaches. This research wishes to answer two research questions:

- RQ1. What can the emerging literature on the sustainability assessments of drone technologies tell us about established methodological approaches?
- RQ2. How should drone products and services be assessed for their sustainability impacts and what are the key considerations?

## Methods

The above research questions guided the research strategy and focus on the collection, screening, and synthesis. The search terms used to limit and capture publications included “LCA & drone”, “Life Cycle Assessment & drone”, “LCA & last mile delivery”,, “LCA & UAV”, “Life Cycle Assessment & UAV”, “Sustainability & drone delivery”, “Sustainability & UAV”, “Sustainability & UAV delivery” “Sustainability & UAV”, “Sustainability & drone”, “Sustainability & last mile drone delivery”, “circularity & drone”, “circularity & last mile delivery”, “circularity & UAV”. The inclusion criteria for the literature were *peer-reviewed journal and conference articles in the English language with a focus on sustainability assessments of drones and drone delivery*. The analysis incorporated a similar analysis to Webster and Watson’s “concept matrix” [10] to record variations in previous sustainability assessments. This involved an analysis of past LCAs conducted, comparing vehicle and type, system boundary, functional unit, drone payload, delivery criteria, and parts production input. Furthermore, industry partners on the MiDrone project were consulted about inputs and primary data collection.

## Assessing the sustainability of drones, delivery systems, and composites

### Life cycle assessment to assess the sustainability of drones

Life Cycle Assessment (LCA) is an analytical tool to measure and compare the environmental impacts of a product, process, system, or service. LCAs are used for decision-making for designers, manufacturers, researchers, and policymakers. The outputs can identify impact hotspots and have the potential to make robust comparisons between different scenarios [11]. LCA studies examine emissions along life cycles or phases: from the extraction of raw materials, transportation, materials processing, manufacturing, distribution, product use, and disposal or recycling at the end of life (EoL) [12] (see Fig. 2). LCA studies separate emissions along life cycles or phases: extraction of raw materials from the earth, transportation, materials processing, manufacturing, distribution, product use, and disposal or recycling at the EoL [12]. LCA studies can be carried out on the entire life cycle such as a cradle-to-grave or a cradle-to-cradle study. Otherwise, LCA studies examine part of a life cycle such as a cradle-to-gate, a gate-to-gate, or a gate-to-grave.

An LCA consists of four iterative phases beginning with the goal definition and scope, followed by Life Cycle Inventory Analysis (LCI), Impact assessment, and interpretation [13]. To define the goal and scope of a product (such as a drone) all the phases in Fig. 2 should be considered. For a drone delivery service study, understanding the operational model of the delivery business and a systems perspective is necessary. Drone delivery services are operated by businesses that are complex and dynamic. A systems approach can capture important interactions and feedback [14]. Furthermore, the functional unit (e.g. a drone or the delivery of 1 kg of) employed in any LCA must align with the goal and scope for suitable comparisons [13].

**Fig. 1:**
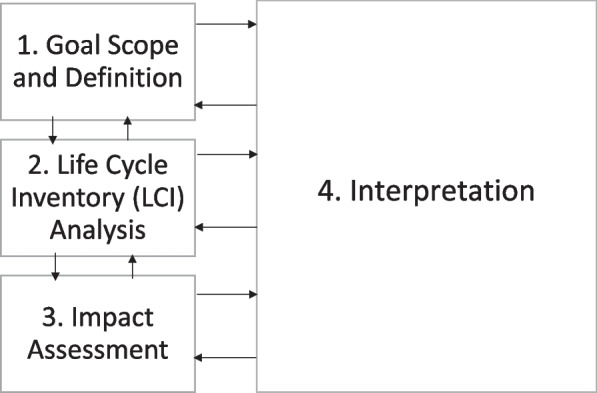
Life Cycle Assessment Framework showing iterations after [13]

A framework to conduct an LCA is detailed in ISO 14040 series and consists of four phases as in Fig. 1 (with an iterative approach) beginning at the goal definition and scope, followed by Life Cycle Inventory analysis (LCI), Impact assessment and interpretation [13]

**Fig. 2:**
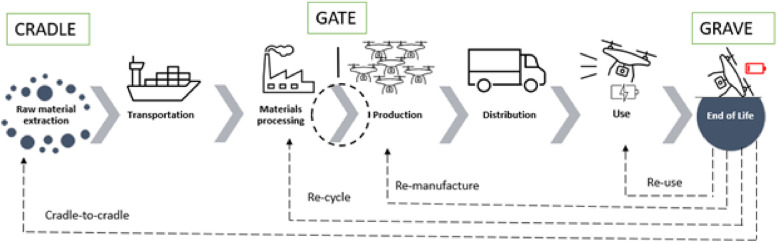
Phases in LCA of a drone to measure impact from raw material extraction to EoL. LCA studies can be conducted from cradle-to-cradle, cradle-to-grave, cradle-to-gate, gate-to-gate, and gate-to-grave depending on the goal and scope of the study

To define the goal and scope of a product (such as a drone) all the phases in Fig. 2 should be considered [13]. To define the goal and scope of a product system, such as a drone delivery service, it is necessary to understand the operational model of the delivery business. A systems thinking perspective is important, as drone delivery services are operated by a business. Businesses are complex and dynamic, and systems thinking can capture important interactions and feedback [14]. The boundaries set at the goal and scope phase draw the limits in the process flow diagram.

The functional unit (FU) is a key element of an LCA as it defines how the study is measured, is aligned with the goal and scope, and allows for appropriate comparisons between systems [13]. For example, an LCA study may compare different scenarios using the FU as one drone unit, or a fleet of drones may be more appropriate. A drone delivery system could be 1 delivery or 100 customer deliveries, and this decision may depend on the operational context. An LCA study compares and considers different scenarios which must be modelled on this FU.

The most critical phase of a robust LCA study is the Life Cycle Inventory (LCI) analysis [15]. This consists of four parts which are: creating a flow diagram, developing a data collection plan, data collection (allocation), and evaluation and reporting. The main approach for compiling an LCI is a process analysis that uses data specific to the product under study. However, due mainly to cost and time constraints, it is impossible to exhaustively assess the entire supply chain of any given product. Data is collected by retrieving primary evidence from companies and/or using established databases (such as Eco Invent) that are continuously updated to reflect evolving realities to increase accuracy [15]. A lack of established data on recycling processes has made conducting comparative LCAs difficult when determining favourable scenarios [16]. There is a lack of quantitative, transparent models for handling aircraft and drones at EoL [17]. The composite materials often end up in landfills, which has led to batteries being a major focus of drone LCAs to date [18]. However, carbon fibre production represents an important aspect of LCA impact categories (such as human toxicity, freshwater aquatic ecotoxicity, marine aquatic ecotoxicity, and terrestrial ecotoxicity) [19], hence more research is needed in this area. Many studies lack transparency in the data. Elements of an LCA are often neglected without convincing evidence to support their exclusion. For example, Bishop et al. [20] recommend that additives should be included in plastic studies when there is no clear proof that their contribution is < 1% to impact categories.

The handling of end-of-life aircraft and UAVs is a relatively undeveloped research topic and little knowledge about the end-of-life process has been reported, as well as a lack of quantitative, transparent models about handling aircraft and drones at the end of their lives [17].

Composites, particularly the most common thermosets, have very little value at EoL so often end up in landfill, which has led to batteries being a major focus of drone LCAs [18]. However, carbon fibre production represents an important aspect of LCA impact categories (such as human toxicity, freshwater aquatic ecotoxicity, marine aquatic ecotoxicity, and terrestrial ecotoxicity) [19, 20]

There is growing area of research activity on the reclamation, reuse, remanufacturing and recycling of composite materials, as activity to develop circular solutions emerges in composite manufacturing research. The University of Bristol has developed the High Performance Discontinuous Fibre (HiPerDiF) Technology to align discontinuous reclaimed carbon fibres (rCFs) through a mechanism that results in a dry fibre preform-tape ready for impregnation with resin. University of Galway has developed a filament for additive manufacturing using recycled plastics and basalt fibres gathered from industrial waste [21]. While this is mainly at a laboratory level, areas of focus are mainly on materials and the retention of mechanical properties, but also on LCA hotspots (where environmental impacts are highest).

There is potential for bio based composites to become a replacement for what is now mostly a fossil-fuel based polymer composite material. Some LCA studies have indicated reduced environmental impacts [22–25] for bio composite materials, but the application of bio composites is in its infancy in most sectors. While there are some promising elements in material performance, it is yet underexplored in UAVs. There are plenty of elements of concern from a life cycle aspect for bio composites (such as durability, moisture, incompatibility and supply chains) yet there are encouraging elements that the data will inform future design for environment strategies [25].

Carbon fibres are particularly important due to the higher economic material value. Basalt fibres have also been investigated such as a study by Ghabezi et al. working on developing additive manufacturing solutions that incorporate in-process waste fibres [21]. There are emerging studies that investigate LCA hotspots which identify the significant environmental impacts in the life cycle. Fitzgerald et al.’s Life Cycle Assessment (LCA) of the HiPerDiF technology (a composite material with discontinuous fibres suitable in aerospace applications) [26]. identified the production of HiPerDiF as an environmental hotspot due to the energy and resources required for the manufacturing process [26].

Many LCAs are conducted on something that is already in existence, and results are often used to support decision-makers. Retrospective LCAs on existing products or services are the most common. However, this may not influence a design change, even though the design is the most important aspect to establish the environmental impact [27]. For emerging technologies, there are different challenges [28]. While drones may seem to be commonplace (for amateur uses), drones for delivery are an emerging technology, as industrial-scale drone delivery services are still in development. There are many challenges with conducting LCAs on emerging disruptive technology. Instincts may tell us a drone is ‘better’ than a car or van, but these new technologies need to prove this environmental advantage with scientific evidence. An ex-ante LCA (as opposed to an ex-post) is recommended by some [28]. Modelling future scenarios, such as full-scale operation, maximum efficiency, use of bio-based materials, and market penetration, can in turn inform design improvement [29]. Cucurachi et al. [28] define ex-ante LCAs as those that.

- “scale-up an emerging technology using likely scenarios of future performance at full operational scale;”
- “compare the emerged technology at scale with the evolved incumbent technology”

The literature on ex-ante LCAs classifies LCA assessments as prospective, consequential, dynamic, anticipatory, and mixed [28]. A dynamic LCA will allow the model to include the temporal aspects so that the LCI is more appropriate. A consequential approach models the significant consequences that may happen as a result of the new technology—such as the removal of another technology e.g. in this instance a fossil-fuelled van or car to replace a drone. There are two main methods to model data: attributional and consequential LCA models. Attributional LCAs are more common in a product system such as a drone delivery service (and all of the studies in this paper are attributional). Consequential LCAs function to include consequences across the different scenarios being modelled [20]. For example, there may be “good” consequences (modelled as a negative value) when a road vehicle is no longer manufactured, used, and scrapped because a drone is now replacing it. However, there are negative consequences to be considered with the increased use and demand of batteries at this time [30].

### Drones, composite materials, and circularity

Government policies and action plans for a circular economy (CE) are driving change in the dominant model of the take-make-waste linear economy. CE offers a framework to improve the sustainability of drones by designing out waste and pollution in all phases, making more durable products to extend the use phase. A CE model can focus attention on designing products for repair, reuse, and remanufacturing as a priority before the consideration of recycling.

CE strategies applied to the making of aerospace parts can displace the historically high “buy-to-fly” ratios (mass of input material required per unit mass of the finished component) in aerospace manufacturing. This ratio is proportional to the high environmental impact at the production stage of the life cycle. This is due to the amount of waste produced in the milling of large block components which evolved to replace the more expensive process of riveting of separate parts together. This ratio is slowly diminishing from a high of 30:1 for Boeing reported in 2005 [31, 31]to more favourable reports in 2018 reporting in-process reuse of titanium which includes 75% scrap material [32]. Cost modelling for aerospace composite applications has emerged due to the high materials costs and increasing structural mass containing high value reinforced composites [33].

Aircraft (containing large volumes of fibre-reinforced composites) are mostly landfilled when they are retired [34]. The impact can depend on manufacturing processes, the low “buy-to-fly” ratio, additives, the carbon intensity of the grid, recycling (and displacing virgin production), and EoL treatment methods [16]. While emissions from landfills are not of concern due to the inert nature of the waste, the Council Directive 1999/31/EC on the “landfill of waste” addresses a reduction of material disposed of by landfill and has a direct impact on composite manufacturers [35]. Incineration produces larger amounts of GHG emissions mainly from the combustion process as the carbon content of carbon fibre is released to the environment as CO_2_ [16].

Composite materials for aircraft are highly challenging in the recovery and recycling phases [35]. Pyrolysis recovery of carbon or glass fibres is positive, as it consumes only 5–10% of the energy required to produce real carbon or glass fibre [36]. However, while composites recycling can reduce impacts, it is often not economically viable [16] outside of non-structural light-weighting applications that do not demand high levels of purity and feedstock quality [16, 37]. A lack of inventory data on recycling processes in life cycle inventory (LCI) databases and in the markets for recycled materials, comparative LCA studies on CFRP recycling are not well established, making it difficult to compare the environmental performance of recycling technologies and determine which one offers the most benefits [16]. The impacts of composites differ greatly due to the different manufacturing processes, additives, carbon intensity of the grid, recyclability, and the EoL treatment methods. The data variation can be so extreme that the carbon footprint of recycled composites can sometimes exceed that of the original production [16].

## Discussion on selected LCA studies of UAV v Ground vehicles

The most comprehensive LCA studies were selected and critically analysed. Table 1 summarises the matrix of concepts and considerations used to make a UAV v ground vehicle comparative analysis. There was a general lack of transparency in the data, however, several important considerations were identified, as discussed in the following sections.

**Table 1:** LCA concept matrix of studies on drones and drone delivery systems

Delivery criteria	System boundary	Functional unit	Used vehicle and type	Drone payload	Parts production input	Ref
One-to-many	Cradle to gate	Transportation of a payload within a 5-mile radius delivering 300 packages per day for two years	Multirotor UAV, Fixed-wing UAV, Truck	1 kg	Multirotor frame, Battery, Charger, Motor, PCB, Transmitter, Optical Sensor	Neuberger (2017) [38]
One-to-one + One-to-many	Cradle to grave: production, operation, disposal	1 package delivered	Multirotor UAV, electrical tricycle, and diesel van	5.0 kg	Battery Vehicle	Figliozzi (2017) [39]
One-to-one	Gate to gate + battery manufacturing for drone and electric vehicle + warehouse emissions	1 package delivered	4 multirotor UAV, 8 multirotor UAV, Personal electric vehicle, Personal car, Gasoline delivery van, Electric truck, Gasoline helicopter drone, Diesel truck, Natural gas truck	0.5 and 8,1 kg	Battery	Stolaroff et. al. (2018) [18]
One-to-one	Gate to gate	1 pizza delivered	Multirotor UAV Motorcycle Electric Motorcycle	-	Out of System boundary	Park et. al. (2018) [40]
One-to-one	Cradle to gate	1 package delivered per km; Lifespan: 5000 h	Multirotor UAV	5 kg	Frame, Servo motor, Cargo box, Propeller, Electronic speed control Batteries	Koiwanit (2018) [19]
One-to-many	Gate to gate	Delivery of parcels to recipient addresses in circular service zones	Multirotor UAV Truck	-	Out of System boundary	Goodchild and Toy (2018) [41]
One-to-one	Cradle to grave	Number of deliveries made by a fleet of 10 UAV in 2 years	Multirotor UAV BEV ICE	20.4 kg	Electricals, Plastic parts, Metal parts, PCB, Battery, Corrugated cardboard	Yowtak et. al. (2020) [42]

### One-to-one v one-to-many considerations

To compete with ground vehicles, a drone delivery company need fleets of drones, not single units [42] which can cause difficulty comparing LCA scenarios. *One-to-one* delivery services are the most common model for food delivery, which is directed by a customer wanting an order within a short time [43]. Ground vehicles for *one-to-one* delivery services are based on their low load capacity, therefore, motorcycles and cargo bikes are an ideal mode, followed by private cars [44]. Stolaroff et al. (2018) *one-to-one* scenario simulated a same-day goods delivery system. The model represents the delivery embedded in a distribution logistics system and incorporates a drone hub [15] Park et al. compared the use phase in a *one-to-one* scenario of drone delivery and a motorcycle [43]. A *one-to-many* service mainly depends on the ground vehicle load capacity and is not usually delivering ready-to-eat food.

### Functional unit considerations

The functional unit has major consequences for the LCA inputs, and there is currently no consistency across key studies. In a *one-to-one* scenario for drones, there is a sequence of depot-costumer-depot. However, since UAV flight depends on a fully charged battery to operate one round trip [45], it is necessary to evaluate the number of batteries the system will require to operate. A well-maintained battery can hold 1000 recharge cycles [46]. The battery lifespan can add more complexity to a drone’s life cycle [39], as more than one drone would have to be included in the system to operate successfully [19].

Park et al. modelled their study on a pizza delivery as the functional unit [40], which is most similar to Figliozzi et al. who used a package delivered (weighing 5 kg) to determine the relative energy efficiency of UAVs. However, Figliozzi et al. examined both *one-to-one* and *one-to-many* models [39]. Goodchild and Toy’s study was based on the electricity for the batteries to receive 1W-hour (Wh) of charge [41]. Yowtak et al. functional unit was based on the drone’s life span, 2 years, carrying 20.4 kg, and worked with a fleet of 10 drones to achieve an equivalent number of deliveries made by a Battery Electrical Vehicle (BEV) without having to recharge [42]. Koiwanit estimated the impacts of a drone delivering a 5 kg package per kilometre with a lifespan of 5000 h equivalent to 250,000 km [19]. Neuberger presented the most comprehensive functional unit for a *one-to-many* delivery system and adapted the functional unit to a time frame of 2 years (drone’s lifespan) [38].

### System boundary considerations

The system boundary considered in these studies is varied, from cradle-to-gate (2 papers), gate-to-gate (3 papers), and cradle-to-grave (2 papers). Only one gate-to-gate study considered parts production deeming lithium-ion batteries as an important portion of the lifecycle impacts of electrified transportation due to manufacturing and raw material extraction [18]. Despite this, there is a consensus among authors that the use phase represents a more significant portion of the overall emissions concerning the environmental impact of UAV delivery systems. The LCA data can be difficult to obtain, as the technology and its applications are quite new. When data is obtained, it is typically acquired from the product manufacturers themselves and is rarely audited [47]. These constraints constitute the barriers researchers face when searching for input/output information during the LCI Hence, current knowledge gaps would result in environmental comparisons with large levels of uncertainty [48].

The use phase comprises what is termed *Generation-to-Propeller* emissions associated with the electricity supply chain including losses in transmission, distribution, charging, and propeller efficiency. For electrical vehicles, the *battery-to-propeller* emissions are considered null whilst for conventional vehicles both *Well-to-Tank* (emissions that take place along the fuel/energy supply chain) and *Tank-to-wheel* (combustion process) are included [39]. The Renewable Energy Directive (EU) 2018/2001 sets a binding target of 32% for renewable energy sources (RES) in the EU’s energy mix by 2030, with a possible review for an increase in 2023 [49]. This target induces a shift in LCA results of electric vehicles. As electricity decarbonises, this use-phase impact decreases [40]. The use of cleaner energy systems, combined with higher battery efficiency, improves the parts production input data, consequently altering the use phase share and its emission proportion [17].

### Temporal dimension considerations

The inclusion of temporal dimension has been an uncommon practice in LCA [50]. Neglecting timing in the analysis can influence outcomes and is related to the functional unit. Varying from one delivery being performed to one day of deliveries, to deliveries made under the drone’s life span and finally, the deliveries made under a motorcycle/truck lifespan can differ significantly in terms of materials input that a poor decision at an early stage of the analysis can potentially lead to bogus outcomes.

## Discussion

This study has revealed a lack of reliable data in past LCA studies, including a lack of robust data regarding parts production and end-of-life phases. None of the studies examined parts production in detail and considerations of composites were absent, and all studies were attributional (which was expected). However, the literature examined has revealed key relationships and dependencies established between the functional unit, the time frame of operation, and the operational model. For this reason, a functional unit suitable for a generic case study should include the transportation of cargo, payload mass, distance covered by the delivery, number of deliveries, and period, which aligns mostly with the Neuberger study [38]. The attention to the particularities associated with the different business models is a key element to establish a fair comparison between UAVs and ground vehicle deliveries.

The industry engagement with the MiDrone project (which provided the context for this study) has revealed the importance of understanding the operations in the manufacture and operation of drones when planning an LCA study. For example, the emissions related to composite manufacturing of airframes may vary considerably by manufacturing facility, such as energy use, waste production, or types of production equipment employed.

The HiPerDiF study highlighted the importance of conducting LCAs for innovative materials and technologiesand can potentially inform future LCAs of discontinuous fibers and other composite materials used in various industries, including lightweight drones [26]

While each of these studies contributes to the growing literature, providing their particular inputs and framework, the multiplicity of approaches taken creates difficulties when comparing different assessments to assess the suitability of each. LCA is very much a user-specific evaluation making a comparison of LCAs on similar topics (like last-mile delivery) complex and regularly impractical [47].

## Conclusion

Key considerations for future studies are; (1) ensure models are like-for-like comparisons by choosing an appropriate functional unit similar to Neuberger [38]; (2) consider a consequential modelling approach when conducting comparative analyses with traditional transport modes to ensure consequences (such as modelling the lower reliance on ICE delivery modes); (3) if additives represent less than 1% of the weight of a product, they should be considered (as suggested by [20] (4) researchers and practitioners should start with simple studies such as gate-to-gate and build from there to allow for high quality data collection and apply appropriate system boundaries and selection of impact categories; (5) keep up to date with emerging technologies in manufacturing, battery, and composite developments; (6) consider the temporal dimension; (7) use primary data for accuracy; (8) for a systems approach ensure the delivery operations are well understood, observations of operations are recommended, and (9) develop new studies to examine circularity to overcome LCA limitation;

This study will inform LCA specialists, manufacturers, and policy-makers on the development and deployment of LCA methodologies and modelling approaches in the achievement of more robust, reliable, transparent, and accurate impact assessment. It has highlighted the challenges to address such as obtaining good data, the uncertainty about future scenario modelling in consequential LCAs, and the lack of studies looking at health or social impacts.

## Data Availability

Data sharing is not applicable to this article as no datasets were generated or analysed during the current study.

## Abbreviations

BEV: Battery Electric Vehicle
CE: Circular Economy
CF: Carbon Fibre
CEAP: Circular Economy Action Plan
CFRP: Carbon Fibre Reinforced Polymer
DTIF: Disruptive Technology Innovation Fund
EoL: End of Life
EU: European Union
FU: Functional Unit
GHG: Greenhouse Gas
HiPerDiF: High Performance Discontinuous Fibre
ICE: Internal Combustion Engine
IPCC: Intergovernmental Panel on Climate Change
LCA: Life Cycle Assessment
LCI: Life Cycle Inventory
PCB: Printed Circuit Board
RQ: Research Question
rCF: Recycled Carbon Fibre
RES: Renewable Energy Source
SDGs: Sustainable Development Goals
UAV: Unmanned Aerial Vehicle
Wh: Watt-hour
